# The Importance of Flare: A Radiological Evaluation of Fixed-Angle Guide and Barrel-Plate in Sliding Hip Screws

**DOI:** 10.7759/cureus.17416

**Published:** 2021-08-24

**Authors:** Sadhin Subhash, Ignatius Liew

**Affiliations:** 1 Orthopaedics, Norfolk and Norwich University Hospitals, Norwich, GBR; 2 Orthopaedics, Addenbrooke's Hospital, Cambridge, GBR

**Keywords:** fracture, neck of femur, sliding hip screw, tip-apex distance, greater trochanter flare

## Abstract

Background and objective

The sliding hip screw (SHS) remains the main operative implant of choice for A1/2 intertrochanteric fractures. These implants are often fixed-angled with a corresponding guide to decrease inventory and implant cost. However, there are varying sizes of base plates on the fixed-angle device between industries. Screw placement is crucial to achieving optimal tip-apex distance (TAD) and position. Due to the flare of the greater trochanter (GT), we hypothesise that the fixed-angle guide can lead to malpositioning. In this study, we aimed to describe the discrepancy between the fixed-angle guide (short: 38 mm, long: 60 mm), the flare of the GT, and the effects on screw placement.

Methods

Patients who received SHS between August to December 2019 were evaluated. We measured the neck-shaft angle, GT flare angle to the femoral axis, screw-plate angle, screw position, and TAD. We templated the optimal 135° fixed-angle barrel-plate, angle guides, and measured the divergence between the angles.

Results

A total of 30 patients were identified to be included in the study; 24/30 (80%) were female, with 16/30 (53%) receiving SHS on the right hip. The average age of the participants was 82 ±9 years. The average neck-shaft angle was 132.4° ±5.9. The GT flare angle was 3.2° ±1.6.

Of note, 66% of patients had a screw-plate angle of ≥135° with an average of 137° ±3.7. However, only 10/30 (33%) screws were placed superiorly, with an average TAD of 21 mm ±11 compared to screws placed in the centre and inferiorly at 9.5 mm ±3 (p=0.0004). The long fixed-angle guide resulted in a lower divergence angle at 3° ±1.7 compared to 5.2° ±2.6 for the short fixed-angle guide (p=0.0001).

Conclusion

Using the fixed-angle guide at 135° on the GT flare results in a sub-optimum screw-plate angle. This can lead to malpositioning of the screw, as well as increased TAD and screw-plate angle. Preoperative planning is crucial to measure the femoral neck-shaft angle, GT flare, as well as utilising a longer fixed-angle guide.

## Introduction

The sliding hip screw (SHS) is the preferred operative implant of choice for A1/2 intertrochanteric fractures [1]. It accounted for 77.8% of all surgical implants used in A1/2 type fractures in the year 2019 according to the National Hip Fracture Database (NHFD) [2]. These implants are often fixed-angle with a corresponding guide to decrease inventory and implant procurement costs. However, the design and length of the guides vary depending on the implant company. Screw placement is crucial to achieving optimal tip-apex distance (TAD) and the position to the femoral neck [2].

The challenges encountered include achieving a TAD of <25 mm to reduce the risk of cut-out, allowing the fracture to impact and collapse, and the task of minimising the complications of metalwork failure such as dissociation of the sliding screw from the barrel [2-3]. The position of the screw is also crucial to reduce the risk of complications with cut-out associated with superiorly placed screws on anteroposterior radiographs [4]. This commonly happens in a mal-reduced fracture in varus, especially with a fixed-angle device such as the SHS at 135°. Within the literature, 3.6% of patients have been reported to face complications that are commonly attributed to the quality of surgical fixation and screw placement [3], with 2.6% of total cases requiring subsequent revision surgery [3].

Due to the flare of the greater trochanter (GT), we hypothesise that the fixed-angle guide can lead to malpositioning. We aim to describe the discrepancy between the fixed-angle guide (short: 38 mm, long: 60mm) [5], the flare of the GT, and the effects on the position of the screw.

## Materials and methods

This was a prospective cohort study evaluating all patients receiving SHS for intertrochanteric A1/2 fractures between August-December 2019 at our institution. The inclusion criteria were all SHS recipients with adequately exposed films pre-op that would allow for templating of the SHS. Exclusion criteria included inadequate or poor quality plain films as well as patients requiring a derotation screw or wire construct on top of an SHS. Patients with any neurological conditions, which resulted in poor quality films, were also excluded from the study.

A total of 30 patients were identified, of which 24/30 (80%) were female, with 16/30 (53%) receiving SHB on the right hip. The average age of our cohort was 82 ±9 years. The average neck-shaft angle was 132.4° ±5.9 on the contralateral side. There were 10/30 (33%) patients with a neck-shaft angle of ≥135° with 4/6 male patients belonging to this category. The GT flare angle was 3.2° ±1.6; 90% (27/30) of the patients had a fracture pattern of A1/2.

The study was assessed for The Research and Ethics Committee approval, which was deemed not required based on the Health Research Authority decision tool on the 10th of December 2019, as this was a service evaluation study.

**Figure 1:**
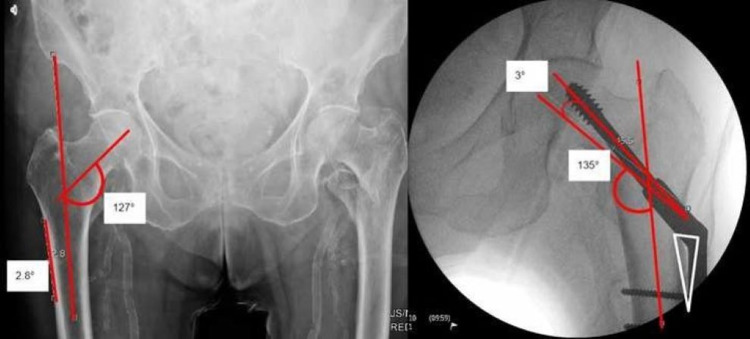
The angle between the femoral anatomical axis and the greater trochanter flare is shown on the left. The screw-plate angle was measured, which was ≥135°, resulting in a ‘sail sign’ when the fixed-angle barrel-plate was placed on the femoral cortex

**Figure 2:**
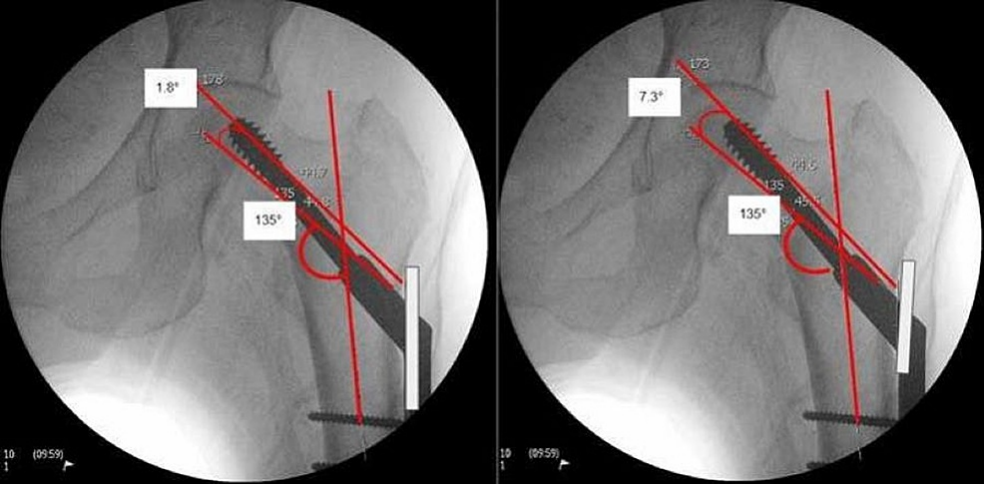
The diverging angles between long (left: 38 mm) and short (right: 60 mm) fixed-angle guide compared to the ideal SHS positioned at 135° to the femoral axis SHS: sliding hip screw

We measured the neck-shaft angle, GT flare angle to the femoral axis, screw position, and TAD. We templated the optimal 135° fixed-angle barrel-plate, angle guides, and measured the divergent angles on post-reduction radiographs using pa 6.7.0.3015 by Agfa HealthCare (Agfa-Gevaert Group, Mortsel, Belgium). The screw-plate angle was measured on intraoperative films. The GT flare angle to the femoral axis was measured, and it is illustrated in Figure 1 (left). The angle between the anatomical axis of the femur and the flat surface of the lateral femoral cortex (starting proximally from the level of the lesser trochanter to illustrate where the SHS base plate guide is commonly placed) was measured. The screw-plate angle was measured, as illustrated in Figure 1 (right). Subsequently, we templated the ideal SHS position with the 135° fixed-angle barrel-plate trajectory. We placed a short 135° (fixed-angle) and long 135° guide from the same entry point, measuring the divergence angles between the trajectory of the guide and the ideal SHS position as illustrated in Figure 2.

The data were tested for normality. The student's t-test was performed on GraphPad Prism version 7.03 (GraphPad Software, Inc, San Diego, CA).

## Results

Of note, 20/30 (66%) patients had a screw-plate angle of ≥135°. In this particular group, it was found that there was an average angle of 137° ±3.7. However, only 10/30 (33%) screws were placed superiorly, with an average TAD of 21 mm ±11 compared to screws placed in the centre and inferiorly at 9.5 mm ±3 (p=0.0004). The long fixed-angle guide resulted in a lower divergence angle at 3° ±1.7 compared to 5.2° ±2.6 for the short fixed-angle guide (p=0.0001). Table 1 presents the results.

**Table 1:** Results table

Screw-plate angle	Number of patients	Percentage (%)
<135°	10	33
>135°	20	66

Direction of screw	Number of screws	Percentage (%)
Superior	10	33
Centre/inferior	20	66

## Discussion

Our study has demonstrated the importance of the GT flare of the femur before performing SHS for intertrochanteric fractures. When utilising a fixed-angle-guide and barrel-plate device, it is important for the surgeon to know about the different implants available, which commonly have an angle of 135°. Preoperative planning, templating, implant availability, as well as patient’s neck-shaft angle, should be considered prior to performing an SHS procedure.

The anatomical flare of the GT significantly affects the guidewire placement. This is evidenced by the short base-plate guides. The longer fixed-angle guide at 60 mm available in the market [DePuy Synthes® (DePuy Synthes, Raynham, MA); Zimmer® (Zimmer Biomet, Warsaw, IN)] corresponds to the GT flare. It provides a lower divergence angle when compared to short fixed-angle guides by Stryker® (Stryker Corporation, Kalamazoo, MI) [5]. This is illustrated in Figure 2; 66% of the patients had a screw placed at ≥135°, which can be attributed to the use of a fixed-angle guide (135°) that is placed flushed on the lateral femoral flare, resulting in an increased angle trajectory of the guidewire beyond 135°. Furthermore, due to the increased angle, as the barrel-plate for the SHS is inserted, it does not lie flush to the femoral cortex, resulting in a subtle ‘sail sign’ as illustrated in Figure 1 (right - ‘sail sign’) due to the screw position at ≥135°.

Hence, it is crucial to achieving a TAD of <25 mm [2], while placing the screw in an inferior (on anteroposterior radiograph) and posterior (on lateral radiograph) position to reduce screw cut-out [3-4]. In a mal-reduced intertrochanteric fracture, a varus neck can lead to superior placement of the screw (30% of our patients, with an average TAD of 21 mm), especially when a fixed-angle guide is used.

There are various methods to reduce the risk of mal-positioning of the screw while incorporating the GT flare of the patient [5]:

1. Preoperative planning is crucial as demonstrated in the literature [6-7]. Prior to surgery, the GT flare angle and the patient’s femoral neck-shaft angle should be measured.

2. Lifting the fixed-angle guide off the lateral cortex distally, to bring the angle of the guidewire placed closer to 135°. This can also be achieved by the freehand technique, which can be unreliable and highly variable depending on experience. However, a better alternative is achieved by abducting the femur on the traction table.

3. Using a variable angle guide only when necessary and starting at 130° as well as having a variety of angles available for barrel-plates.

4. In patients with naturally varus neck-shaft angles, placing the entry point inferiorly is crucial to achieving a TAD of <25 mm [8].

Within the A1/2 intertrochanteric proximal femoral fractures, the SHS construct remains the implant of choice for various reasons, including lower cost and decreased mortality compared with cephalomedullary devices based on the NHFD [9-10]. We have demonstrated the importance of intraoperative screw placement besides presenting technical tips to improve this. Risk factors of failure in the SHS construct are multifactorial, including TAD, screw position, quality of reduction, fracture pattern, and patients' age [11].

The technical limitations of this study include a small sample size and the exclusion of the patients with poor quality plain radiograph imaging, as well as a lack of clinical correlation of patients with inadequate screw position and placements. All patients had an SHS performed and/or supervised by a senior orthopaedic surgeon.

## Conclusions

Using the fixed-angle guide at 135° on the GT flare results in a sub-optimum screw-plate angle. This can lead to malpositioning of the screw as well as increased TAD and screw-plate angle. In this study, we have demonstrated the resultant increase in the divergent angle of 5.2° by using a short base-plate, compared to a long base-plate, which gives an angle of 3°. Preoperative planning is crucial to measure the femoral neck-shaft angle, GT flare, as well as utilising a longer fixed-angle guide.

## References

[1] (2021). National Hip Fracture Database (NHFD) Annual Report 2019. https://www.nhfd.co.uk/files/2019ReportFiles/NHFD_2019_Annual_Report_v101.pdf.Access3rdMarch.

[2] Baumgaertner MR, Curtin SL, Lindskog DM, Keggi JM (1995). The value of the tip-apex distance in predicting failure of fixation of peritrochanteric fractures of the hip. J Bone Joint Surg Am.

[3] Chirodian N, Arch B, Parker MJ (2005). Sliding hip screw fixation of trochanteric hip fractures: outcome of 1024 procedures. Injury.

[4] Pervez H, Parker MJ, Vowler S (2004). Prediction of fixation failure after sliding hip screw fixation. Injury.

[5] Sheharyar K, Newton AW, Harrison WJ (2020). Length matters: short base-plate angle guides may lead to guide-wire mal-positioning when inserting a DHS. A radiographic modeling study. J Orthop Res.

[6] Alao U, Liew I, Yates J, Kerin C (2018). Correlation between the length from the elbow to the distal interphalangeal joint of the little finger and the length of the intramedullary nail selected for femoral fracture fixation. Injury.

[7] Liew I, Qureshi M, Joseph J, Bailey O (2017). Novel technique to accurately measure femoral diameter using a Thomas splint. Injury.

[8] Simpson AH, Varty K, Dodd CA (1989). Sliding hip screws: modes of failure. Injury.

[9] Whitehouse MR, Berstock JR, Kelly MB, Gregson CL, Judge A, Sayers A, Chesser TJ (2019). Higher 30-day mortality associated with the use of intramedullary nails compared with sliding hip screws for the treatment of trochanteric hip fractures: a prospective national registry study. Bone Joint J.

[10] Mellema JJ, Janssen S, Schouten T, Haverkamp D, van den Bekerom MP, Ring D, Doornberg JN (2021). Intramedullary nailing versus sliding hip screw for A1 and A2 trochanteric hip fractures. Bone Joint J.

[11] De Bruijn K, den Hartog D, Tuinebreijer W, Roukema G (2012). Reliability of predictors for screw cutout in intertrochanteric hip fractures. J Bone Joint Surg Am.

